# Sex-specific differences in resting-state functional connectivity of large-scale networks in postconcussion syndrome

**DOI:** 10.1038/s41598-020-77137-4

**Published:** 2020-12-15

**Authors:** Reema Shafi, Adrian P. Crawley, Maria Carmela Tartaglia, Charles H. Tator, Robin E. Green, David J. Mikulis, Angela Colantonio

**Affiliations:** 1 Rehabilitation Sciences Institute, University of Toronto, 160-500 University Avenue, Toronto, ON M5G 1V7 Canada; 2 KITE-Toronto Rehabilitation Institute, University Health Network, Toronto, ON M5G 2A2 Canada; 3 Department of Medical Imaging, Toronto Western Hospital, 399 Bathurst St., Toronto, ON M5T 2S8 Canada; 4 Institute of Medical Sciences, University of Toronto, Toronto, ON M5S 1A8 Canada; 5 Tanz Centre for Research in Neurodegenerative Diseases, University of Toronto, Krembil Discovery Tower, 60 Leonard Ave, Toronto, ON M5T 0S8 Canada; 6 Canadian Concussion Center, Toronto Western Hospital, 399 Bathurst St., Toronto, ON M5T 2S8 Canada; 7 Division of Neurology, Krembil Neuroscience Centre, Toronto Western Hospital, 399 Bathurst St., Toronto, ON M5T 2S8 Canada; 8 Division of Brain, Imaging and Behaviour-Systems Neuroscience, Krembil Neuroscience Centre, Toronto Western Hospital, 399 Bathurst St., Toronto, ON M5T 2S8 Canada; 9 Department of Surgery, University of Toronto, 1 King’s College Circle, Toronto, ON M5S 1A8 Canada; 10 Division of Neurosurgery, Krembil Neuroscience Centre, Toronto Western Hospital, 399 Bathurst St., Toronto, ON M5T 2S8 Canada; 11 Department of Occupational Science and Occupational Therapy, Dalla Lana School of Public Health, University of Toronto, Toronto, ON Canada

**Keywords:** Neuroscience, Physiology, Brain injuries

## Abstract

Concussions are associated with a range of cognitive, neuropsychological and behavioral sequelae that, at times, persist beyond typical recovery times and are referred to as postconcussion syndrome (PCS). There is growing support that concussion can disrupt network-based connectivity post-injury. To date, a significant knowledge gap remains regarding the sex-specific impact of concussion on resting state functional connectivity (rs-FC). The aims of this study were to (1) investigate the injury-based rs-FC differences across three large-scale neural networks and (2) explore the sex-specific impact of injury on network-based connectivity. MRI data was collected from a sample of 80 concussed participants who fulfilled the criteria for postconcussion syndrome and 31 control participants who did not have any history of concussion. Connectivity maps between network nodes and brain regions were used to assess connectivity using the Functional Connectivity (CONN) toolbox. Network based statistics showed that concussed participants were significantly different from healthy controls across both salience and fronto-parietal network nodes. More specifically, distinct subnetwork components were identified in the concussed sample, with hyperconnected frontal nodes and hypoconnected posterior nodes across both the salience and fronto-parietal networks, when compared to the healthy controls. Node-to-region analyses showed sex-specific differences across association cortices, however, driven by distinct networks. Sex-specific network-based alterations in rs-FC post concussion need to be examined to better understand the underlying mechanisms and associations to clinical outcomes.

## Introduction

The long-term effects of traumatic brain injury (TBI) can have lifelong consequences on health and wellbeing leading to poor recovery outcomes^1,2^. Despite lack of standardized monitoring, early symptom resolution and underreporting (especially within the sports and assault contexts), mild TBI (mTBI) is estimated to account for 75–90% of all TBI^3^. It is estimated that 15% of individuals will continue to report persistent symptoms for more than 3 months post concussion^4,5^. Symptoms may persist for months to years post injury and may be permanent in some cases^6^. mTBI can result in a wide range of physical, cognitive, psychological and social impairments^7,8^ that affect a person’s ability to recover post injury and resume life roles and activities of daily living, such as return to play or work.

Resting state functional magnetic resonance imaging (rs-fMRI) has reliably been used to investigate network connectivity and neural organization in-vivo. There is growing evidence that the human brain is intrinsically organized into distinct functional networks that support complex mental processing^9–12^ linked to cognitive function and behavior. In recent years, three large-scale resting state brain networks have received considerable attention as they provide a common framework to understand dysfunction across multiple disorders, especially related to cognition^13^: the default-mode network (DMN), the salience network (SN), and the fronto-parietal network (FPN). The DMN spans the largest surface across the brain and is associated with supporting self-referential mental activity including autobiographical and self-monitoring functions^14,15^. The SN, anchored in the insula and anterior cingulate cortex, is thought to be involved in the detection, identification and filtering of the most homeostatically relevant internal and external stimuli to guide behavior^16,17^. The lateralized FPN is thought to play a role in influencing hierarchical cognitive processes such as attention, memory and goal-directed behavior^18,19^. The dynamic synchronization of these networks^20^ offers a unique opportunity to study complex network dynamics in the resting state.

Alterations in these three neural networks after TBI of various severity have been documented in the literature^21–23^ and a large number of studies have explored network-related changes during the acute and sub-acute stages after concussion^24–26^. There are, however, only a small number of studies exploring the alterations in network-related connectivity in postconcussion syndrome (PCS). Although there are several definitions of PCS as was discussed recently^27^, the criteria for a widely used definition include a history of TBI, cognitive deficits, presence of three of eight TBI symptoms (fatigue, sleep disturbance, headache, dizziness, irritability, affective disturbance, personality change, apathy) that began post-injury and persisted for ≥ 3 months post injury and are associated with social function interference^28^. We and others have previously used a month criterion^29^.

While males may be more prone to sustaining a brain injury^30^, emerging evidence suggests that females have worse outcomes on 85% of the TBI indicators when compared to men and older females have higher vulnerability for development of prolonged PCS^31–33^. However, there is a dearth of studies examining sex-based differences both in functional and structural connectivity post concussion. A 2014 review paper^34^ included 122 publications exploring neuroimaging findings in mTBI over a 21 year period (1990–2011) yet none of these studies had explored sex-based differences post mTBI nor did the review identify this lack of exploration as a gap in the literature. More recent studies of neuroimaging provide some evidence for sex-based differences in working memory functional activation circuitry post concussion^35^, measures of aggression and orbitofrontal functional connectivity patterns^36^ as well as structural connectivity^37,38^. Despite this emerging evidence, to date there are no studies investigating sex differences in network based connectivity alterations following PCS. For this study, our aim was to investigate the network based changes across three large scale networks (DMN, SN and FPN) as well as network-to-regional connectivity to determine the sex-based differences post concussion.

## Methodology

### Participants

All subjects who attended the Neurology Clinic at the Toronto Western Hospital from April 2013 to October 2015 were screened for this study. A total of 129 participants met the following inclusion criteria: 16–60 years of age; had at least one concussion; persistent symptomatology since the last concussion; and at least one month post concussion. Exclusion criteria were as follows: pre-existing brain disease; brain injury more severe than concussion; positive MRI findings; MRI contraindicated or of poor image quality. A total of 111 participants were included in the study. Control participants had no known neurological and/or psychiatric disorder. The study was approved by the University Health Network Research Ethics Board at the Toronto Western Hospital and all procedures were conforming to standards set by the latest revision of the Declaration of Helsinki. All participants provided written informed consent to participate.

### MRI acquisition

Participants underwent MRI scans on a 3 T MRI scanner (GE Healthcare, Signa HDx, Milwaukee, WI, USA) fitted with a standard 8-channel phased array head coil to obtain high resolution structural images and resting state functional MRI (rs-fMRI) images. A high resolution 3D T1-weighted image was obtained using inversion recovery preparation pulse fast 3D gradient echo^39^. The parameters were: 180 axial slices with 1 mm thickness; 2.6-ms echo time (TE); 6.9-ms repetition time (TR); 450-ms inversion time (TI); 15° flip angle; 256 × 256 matrix size; 1 × 1 × 1 mm voxel size. The rs-fMRI data were acquired using a single-shot gradient echo EPI sequence, with 45 axial slices with 3.5 mm thickness; TE = 25 ms, TR = 2.5 s; 64 × 64 matrix; 3.5 × 3.5 × 3.5 mm voxel size; parallel imaging (ASSET). During the scans, participants were instructed to rest with their eyes closed.

### Resting-state fMRI pre-processing and data analysis

The anterior commissure origin was set for images using AFNI. The CONN-fMRI Functional Connectivity toolbox (ver.17; https://www.nitrc.org/projects/conn) using SPM8 (https://www.fil.ion.ucl.ac.uk/spm/) was used to process resting state data^40^. Images underwent a default pipeline which included functional realignment and unwarp, slice-timing correction, structural segmentation and normalization, functional normalization, ART-based functional outlier detection and scrubbing, and functional smoothing (8-mm Gaussian kernel) carried out in MNI-space. Functional connectivity maps were calculated for within and between networks (node-to-node) as well as network-to-region. Individual connectivity maps were created for each participant. A total of fifteen nodes were selected for the three networks of interest. For the DMN, the medial prefrontal cortex (mPFC) and the posterior cingulate (PCC) and the bilateral lateral parietal (LP) regions were selected as nodes; for the SN, the anterior cingulate cortex (ACC) along with the bilateral anterior insulae (AI), rostral prefrontal cortices (RPFC) and supramarginal gyri (SMG) served as nodes; while the dorsolateral prefrontal cortex (DLPFC) and the posterior parietal cortex (PPC) bilaterally served as the nodes for the FPN.

### Connectivity matrix for network- and region-based analysis

To investigate group differences in rs-FC between the three large scale networks of the brain, a symmetrical 15 × 15 connectivity matrix was computed for each subject. For each pair of nodes, a connectivity value was calculated as the Pearson correlation between the average time-courses for each node, after partialling out any correlation with white matter and CSF time-courses using the aCompCor procedure^41,42^ and head-motion covariates (calculated during functional realignment). Each correlation value was then converted to a Fisher z-score which we refer to as connection strength. Refer to Table 1 for network assignment and co-ordinate information. To control for multiple comparisons, we used the network based statistic (NBS) approach^43,44^ to control for family-wise error rate. NBS is a nonparametric cluster-level statistics technique that defines “clusters” using the graph theoretical concept of connected components. NBS has been used to control for family-wise errors when performing mass univariate testing. NBS was chosen given its advantage of rejecting the null hypothesis at the network levels which allows one to observe the effect of significant network clusters rather than significant individual connections. Both the T statistic and the corresponding p-value for the contrast of interest were reported.

**Table 1:** Network assignment and coordinates for network nodes.

Network nodes	Coordinates (x, y, z)
**Default mode network**	
Medial prefronal cortex	1, 55, − 3
Lateral parietal (right)	47, − 67, 29
Lateral parietal (left)	− 39, − 77, 33
Posterior cingulate cortex	1, − 61, 38
**Salience network**	
Rostral prefrontal cortex (right)	32, 46, 27
Rostral prefrontal cortex (left)	− 32, 45, 27
Anterior cingulate cortex	0, 22, 35
Anterior insula (right)	47, 14, 0
Anterior insula (left)	− 44, 13, 1
Supramarginal gyrus (right)	62, − 35, 32
Supramarginal gyrus (left)	− 60, − 39, 31
**Fronto-parietal network**	
Lateral prefrontal cortex (right)	− 32, 45, 27
Lateral prefrontal cortex (left)	41, 38, 30
Posterior parietal cortex (right)	52, − 52, 45
Posterior parietal cortex (left)	− 46, − 58, 49

To utilize NBS for each group difference of interest, the differences in connection strengths were thresholded at an uncorrected p < 0.05 to form a “cluster” of suprathreshold connections. The cluster was then assessed for significance based on the intensity i.e. the sum of connection strengths, while permutation testing was used to control the rate of falsely detecting networks at pFWE < 0.05.

Finally, we explored network-to-region rs-FC using both false discovery rate (FDR)-correction as well as NBS. A 15 × 133 connectivity matrix was created, in which each element represents a connection strength between an a priori node and one of CONN’s 133 regions of interest (ROI) created by the software’s default parcellation scheme. We employed FDR control (pFDR < 0.05) for each of the 15 nodes separately. When the null hypothesis is true for any given node, FDR control reverts to a weak FWE control (pFWE < 0.05) over the total number of possible connections to that node. From the binomial expansion of total probability, the probability of one false positive for such a node analysis is approximately 0.05 and the probability of 2 false positives is approximately 0.001. Therefore to a good approximation, the overall FDR across all 15 nodes is well controlled by ignoring the results of any nodes that yield only one positive result. NBS was also used to find significant clusters of connection differences over the same matrix.

Group differences for rs-FC in both between networks and network-to-region connection strengths were explored between concussed and control groups, males and females as well as for the interaction effect of sex by injury. Age was included as a regressor in the model to account for age-related variability between sub-groups of interest.

## RESULTS

### Study sample

The sample consisted of 80 individuals with postconcussion syndrome, 47 males with mean age ± SD = 32 ± 13 years and 33 females with mean age ± SD = 31.8 ± 13.1 years. The 31 controls consisted of 17 males with mean age ± SD = 39.5 ± 10.4 years and 14 females with mean age ± SD = 32.3 ± 14.1 years. The demographic characteristics are presented in Table 2. There were no significant differences between male and female concussed groups when comparing time-lapsed since injury and/or number of concussions.

**Table 2:** (a) Participant demographics; subjects by injury and sex (n = 111) and (b) Concussion-specific features and reported symptomatology (n = 80).

2a	Concussed group (n = 80)		*p*-value (effect size)	Control group (n = 31)		*p*-value (effect size)
Number of participants	Males (n = 47)	Females (n = 33)		Males (n = 17)	Females (n = 14)	
Age in years (mean, SD)	32.49	31.82	0.93	39.53	32.28	0.39
	± 12.73	± 13.12	(0.05)	± 10.42	± 14.11	(0.20)
Education in years (mean, SD)	14.47	15.00	0.92	16.33	16.00	0.95
	± 2.43	± 2.99	(0.20)	± 3.20	± 2.10	(0.03)
# of prior concussions (mean, SD)	4.36	2.66	0.52	–	–	*–*
	± 3.11	± 1.51	(0.73)			
Time lapsed since concussion in months	18.79	20.70	0.76			
	± 21.28	± 29.37	(0.08)			

2b						
	n (%)			n (%)		
**Treatment-related**			**Medical and psychological**			
Reported loss of consciousness	14 (30%)	10 (30%)	Headaches	42 (89%)	31 (94%)	
Reported post-traumatic amnesia	9 (19%)	7 (21%)	Dizziness	37 (79%)	28 (85%)	
Visited ER	20 (43%)	15 (45%)	Fatigue	30 (64%)	24 (73%)	
Hospitalized	5 (11%)	4 (12%)	Photophobia	29 (62%)	21 (64%)	
Consulted family physician	21 (45%)	19 (58%)	Balance difficulties	28 (60%)	15 (45%)	
			Phonophobia	24 (51%)	19 (58%)	
Depression	29 (62%)	15 (45%)	Persisting pain	19 (40%)	13 (39%)	
Anxiety	27 (57%)	21 (64%)	Blurred vision	15 (32%)	10 (30%)	
Irritability	34 (72%)	24 (73%)	Other neurological symptoms	42 (89%)	31 (94%)	

#### Injury-based analysis (PCS vs. controls)

Mean BOLD rs-FC showed good identification of the three intrinsic connectivity networks, namely DMN, SN and FPN. This is consistent with reports in the literature^45^.

### Within and between network connectivity analysis (NBS)

**Figure 1:**
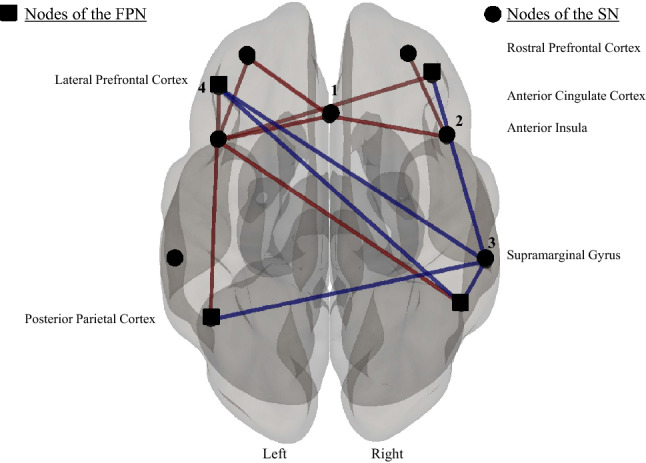
Seed-to-seed functional connectivity using Network Based Statistics (p-FWE = 0.04). Red lines represent increased rs-FC while blue lines represent reduced rs-FC. The sphere represent nodes of the SN while the squares represent the nodes for the FPN. Image is displayed using neurological convention. Contrast image concussed > controls, shows that concussed participants showed significantly increased connectivity in the frontal nodes including the anterior insula and significantly reduced connectivity amongst the lateral prefrontal and parietal nodes when compared to control participants. 1 = Anterior Cingulate Cortex; 2 = Anterior Insula; 3 = Supramarginal Gyrus; 4 = Lateral Prefrontal Cortex. Image generated using CONN, an open- source computational platform available at https://web.conn-toolbox.org/ home.

Group differences both within and between networks were explored using NBS. On the connectivity contrast (concussed versus controls), most of the SN and all of the FPN a priori nodes were functionally connected as a single network based on strength of the connections (p-FWE = 0.04). Concussed participants showed a visibly hyperconnected frontal SN component with a hypoconnected parietal SN component when compared to control participants (see Fig. 1). More specifically, the left AI of the SN showed increased within network rs-FC to the two frontal nodes of the SN (the ACC and the left RPFC) as well as all four nodes of the FPN while the right SMG node of the SN showed reduced connectivity to all four nodes of the FPN with no significant influence with the SN. No statistically significant differences were observed within or between DMN rs-FC between concussed and control participants.

### Network-to-region connectivity analysis (NBS)

Group differences in rs-FC between the a priori network nodes and brain regions were also explored using NBS (see Fig. 1). When comparing concussed and control participants, we observed altered rs-FC based on connection strength, again across the same prominent nodes of the network, the right SMG (p-FWE = 0.03) and the left AI of the SN (p-FWE = 0.06); the latter only nearing significance. While both these nodes showed distributed rs-FC in a contrast of group differences, the right SMG had a predominantly increased connectivity across 29 regional sites, largely across the temporal and opercular cortices but reduced connectivity to frontal regions. Similarly, the left AI had increased rs-FC across 28 regional sites predominantly across the frontal and parietal regions with reduced rs-FC in the right cerebellum region (see Table 3).

**Table 3:** Node-to-region connectivity differences between concussed and control participants using network based statistic.

Node-to-region connectivity	Statistic	p-FDR
**SN node: right supramarginal gyrus**		
Planum temporale left	3.58	< 0.001
Parietal operculum cortex right	3.49	< 0.001
Central opercular cortex right	3.48	< 0.001
Heschl's gyrus left	3.43	< 0.001
Juxtapositional lobule cortex right	3.14	0.002
Planum temporale right	3.08	0.003
Precentral gyrus right	3.08	0.003
Superior temporal gyrus, posterior division right	3.01	0.003
Middle frontal gyrus right	− 2.99	0.004
Juxtapositional lobule cortex left	2.94	0.004
Central opercular cortex left	2.93	0.004
Inferior temporal gyrus, temporooccipital part right	− 2.83	0.006
Heschl's gyrus right	2.82	0.006
Temporal pole right	2.68	0.009
Insular cortex right	2.52	0.013
Superior temporal gyrus, anterior division right	2.47	0.015
Amygdala right	2.45	0.016
Precentral gyrus left	2.44	0.016
Planum polare left	2.42	0.017
Planum polare right	2.36	0.020
Frontal pole right	− 2.32	0.022
Cerebelum lobule × left	2.29	0.024
Cuneal cortex right	2.17	0.032
Frontal pole left	− 2.14	0.035
Superior temporal gyrus, posterior division left	2.09	0.039
Intracalcarine cortex left	1.98	0.050
**SN node: left anterior insula**		
Inferior frontal gyrus, pars triangularis right	3.53	< 0.001
Inferior frontal gyrus, pars opercularis right	3.15	0.002
Middle frontal gyrus left	2.98	0.004
Inferior frontal gyrus, pars opercularis left	2.87	0.005
Caudate right	2.86	0.005
Angular gyrus right	2.77	0.007
Precentral gyrus left	2.65	0.009
Angular gyrus left	2.59	0.011
Caudate left	2.57	0.012
Juxtapositional lobule cortex left	2.53	0.013
Supramarginal gyrus, posterior division left	2.48	0.015
Inferior frontal gyrus, pars triangularis left	2.48	0.015
Frontal pole left	2.42	0.017
Cerebelum lobule × right	− 2.38	0.019
Pallidum left	2.35	0.021
Accumbens left	2.32	0.022
Juxtapositional lobule cortex right	2.13	0.036
Precentral gyrus right	2.11	0.037
Supramarginal gyrus, posterior division right	2.08	0.040
Cerebelum lobule VII right	2.05	0.042

### Network-to-region connectivity analysis (FDR)

Exploring group differences in rs-FC between the fifteen a priori network nodes and the 133 ROIs using FDR-correction, revealed increased rs-FC in the concussed group when compared to the control group between the (1) right SMG and the right central opercular cortex, the right parietal opercular cortex, the left Heschl’s gyrus, and the left planum temporale (p = 0.04) as well as (2) right insula and the right inferior frontal gyrus, pars triangularis (p = 0.02).

#### Sex- and injury-specific analyses

**Figure 2:**
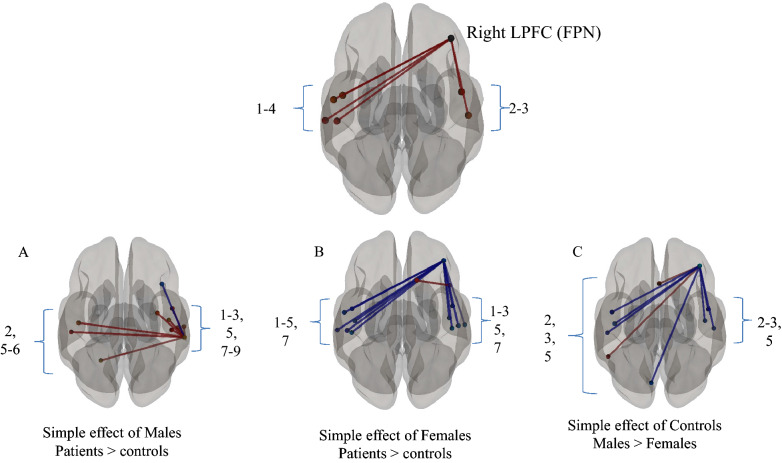
**-** The interaction effect of injury (males > females) × injury (concussed > controls) as well as the simple effects of (**A**) males, (**B**) females and (**C**) controls. The images are displayed using neurological convention. 1. Superior Temporal Gyrus, Posterior division; 2. Planum Temporale; 3. Central Opercular Cortex; 4; Lateral sensorimotor network; 5. Heschl's Gyrus; 6. Cerebellar lobule V1; 7. Parietal Opercular Cortex; 8. Pre Central Gyrus; 9. Middle Frontal Gyrus; 10. Subcallosal Cortex; 11. Inferior Temporal Gyrus, temporooccipital part; 12. Cuneal Cortex. Image generated using CONN, an open- source computational platform available at https://web.conn-toolbox.org/home.

**Figure 3:**
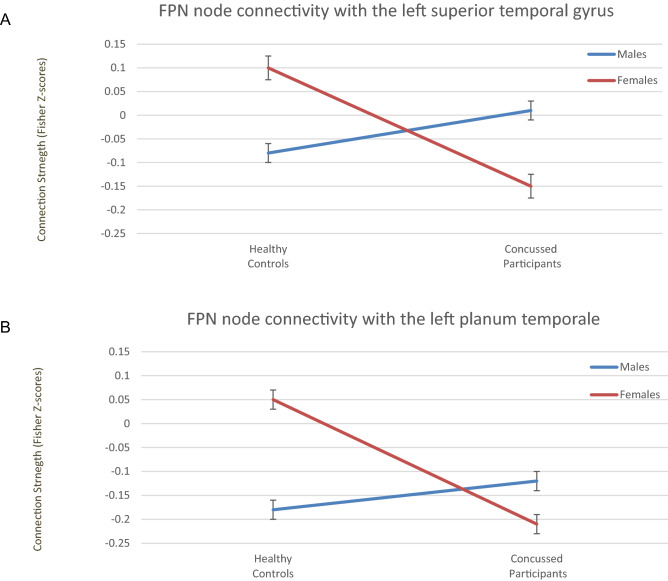
Sex (males > females) × injury (concussed > controls) interaction for seed-to-network connectivity of the right lateral prefrontal cortex, a node of the FPN, with (**A**) the left superior temporal gyrus (posterior division) and (**B**) the left planum temporale.

Sex-based differences in rs-FC between network nodes and brain regions were also explored using NBS, however, an interaction term (sex × injury) was not significant based on the strength of the network connections. Following FDR correction, however, an interaction effect was significant for the right LPFC node of the FPN and the bilateral temporal/opercular regions. These regions included the left superior temporal gyrus, the central opercular cortices bilaterally, the planum temporale bilaterally and the left lateral sensorimotor network (see Table 4). Post hoc analyses revealed that while in the control group females had increased rs-FC between the right LPFC of the FPN and temporal/opercular regions (p < 0.002) compared to males, in the females-only sample concussed females had reduced connectivity in these and adjacent regions when compared to the control females (p < 0.002). Concussed males, however, had increased connectivity to these regions compared to male controls (see Figs. 2 and 3), albeit through the right supramarginal gyrus.

**Table 4:** Injury by sex interaction effects of node-to-region connectivity.

Contrast	Node-to-region connectivity	Statistic	p-FDR
**Interaction effect**	**Right LPFC of the FPN**		
	Planum temporale left	4.79	< 0.001
	Superior temporal gyrus, posterior division left	4.73	< 0.001
	Planum temporale right	3.71	< 0.001
	Central opercular cortex left	3.68	< 0.001
	Central opercular cortex right	3.47	< 0.001
	Lateral sensorimotor left	3.42	< 0.001
**Simple effect of controls**	**Right LPFC of the FPN**		
Females > males	Planum temporale left	4.05	< 0.001
	Central opercular cortex left	3.79	< 0.001
	Central opercular cortex right	3.52	< 0.001
	Planum temporale right	3.36	0.001
	Heschl's Gyrus Left	3.28	0.001
	Heschl's gyrus right	3.17	0.002
	Cuneal cortex left	3.12	0.002
Males > Females	Subcallosal cortex	3.31	0.001
	Inferior temporal gyrus, temporooccipital part left	3.27	0.002
**Simple effect of females**	**Right anterior insula**		
Concussed > controls	Right superior frontal gyrus	3.82	< 0.001
Controls > concussed	**Right LPFC of the FPN**		
	Planum temporale left	5.35	< 0.001
	Superior temporal gyrus, posterior division left	4.85	< 0.001
	Central opercular cortex left	4.35	< 0.001
	Heschl's Gyrus Left	4.10	< 0.001
	Lateral sensorimotor left	3.76	< 0.001
	Heschl's gyrus right	3.69	< 0.001
	Planum temporale right	3.63	< 0.001
	Central opercular cortex right	3.57	< 0.001
	Parietal opercular cortex left	3.36	0.001
	Superior temporal gyrus, posterior division right	3.31	0.001
	Parietal opercular cortex right	3.24	0.002
**Simple effect of males**	**Right supramarginal gyrus**		
Concussed > controls	Planum temporale left	4.38	< 0.001
	Parietal opercular cortex right	4.00	< 0.001
	Central opercular cortex right	3.74	< 0.001
	Planum temporale right	3.71	< 0.001
	Superior temporal gyrus, posterior division right	3.58	< 0.001
	Heschl's gyrus left	3.55	< 0.001
	Precentral gyrus right	3.13	0.002
	Heschl's gyrus right	3.12	0.002
	Cerebelum lobule VI right	3.08	0.003
Controls > concussed	Middle Frontal gyrus right	3.14	0.002

## Discussion

To the best of our knowledge, this is the first study that has explored neural alterations in BOLD rs-FC across three large scale networks to provide evidence regarding sex-specific changes in PCS.

First, we provide evidence that concussed individuals have altered rs-FC between and within two large scale networks, the SN and the FPN. In concussed participants, these alterations are manifested as increased rs-FC within the fronto-insular nodes and reduced rs-FC between the parietal node of the SN and both frontal and parietal nodes of the FPN when compared to age- and sex-matched controls.

Our overall findings of increased rs-FC across the SN and FPN in the chronic stages post-concussion are largely consistent with others that have explored rs-FC across similar injury severities and stages of recovery (3 months or more). At 6 months post-concussion, an overall increase in network-to-regional rs-FC, especially between the ACC/DLPFC and the frontal, parietal and temporal regions of the brain, has been reported^46^. In their study, Czerniak et al. (2015) did not explore node-based network connectivity thus we are unable to compare the network-based alterations observed in our sample.

Second, using NBS, we report that concussed individuals have altered rs-FC across two SN nodes (right SMG and left AI) and with the frontal, parietal and temporal regions of the brain, with increased connectivity to temporal and reduced connectivity to frontal and parietal regions. In particular, AI connectivity was increased with the inferior and middle frontal gyri. There is support that the short intralobular fronto-insular U-tract system directly connects the anterior insula to frontal regions including the right IFG^47^. It is thought that these frontal regions play a generalized role in executive functions (attention, working memory etc.) and have shown atrophy after severe brain injury^48–50^.

Third, we describe an interaction effect of injury by sex. Concussed females showed a statistically significant reduction in rs-FC of FPN node (right LPFC) to the contralateral temporal and opercular cortices relative to both concussed males and controls. Our post-hoc analyses indicated that female controls have an increased rs-FC between the right LPFC (FPN) and bilateral temporal/opercular cortices compared to males, however, following concussion females show a reduced connectivity to the contralateral regions when compared to concussed males as well as controls. This interaction is only seen when females are included in the contrast suggesting a female “vulnerability” in the FPN post-concussion. Additionally, in a males-only sample, concussed participants also had an increased rs-FC within similar temporal and opercular cortices across both the ipsi- and contra-lateral cortices when compared to controls. This alteration, however, was driven by the SMG (SN) and not the LPFC (FPN) as seen with females. This sex-dependent SMG connectivity alteration is only observed as a simple effect of injury in males. The LPFC plays a distinct role in differentiating amongst salient information to establish a set of rules that focus resources on the defined goal related to the task-at-hand^49^. The reduced rs-FC in concussed females between the right LPFC and, for instance, the superior temporal gyrus (STG) may imply an underlying failure to reliably recruit certain cortical association regions for information processing. Certain regions of the temporal cortices are considered to be sexually dimorphic given known hemispheric and network asymmetries^50,51^. Males have a more pronounced leftward asymmetry in certain cortical regions^52^ including the planum temporale (PT), STG and the parietal operculum (PO) cortex when compared to females. Females have a hemisphere dependent specialization in neural organization in the right PT but not left when compared to males^53^. Also, the STG has been implied to play a role in functional switching of auditory attention, commonly referred to as the cocktail party effect^54^. Amongst cognitive impairments, attentional disruptions and working memory impairments are commonly reported after brain injury^55,56^.

We propose that these network-level alterations represent a hypervigilant state or a ‘salience interference effect’ in PCS. It has been suggested that the SN integrates information through its connections with cortical and sub-cortical sites^57^. In the event of a salience detection, the SN recruits the FPN to manipulate and process information in a sustained manner across various cortical and sub-cortical hierarchical circuits to facilitate goal-directed behavior^16,58^. We propose that this resting-state alteration within the hubs of the SN may be responsible for taxing the circuitry and impeding the FPN’s ability to efficiently tap into association cortices thus leading to interference with hierarchical neural processing and hence goal-directed tasks. Using a context-driven approach^59^, we interpret this hypervigilent state post concussion within the framework of underlying cytoarchitecture. The fronto-insular cortex and the ACC regions have a distinct morphological advantage with the presence of the Von Economo neurons (VEN) in layer V of the cortex^60^. Given their noradrenergic input^61^, their unique morphology by way of their extended projections, scarce dendritic spines^62^ and diverse cortical and suborbital targets^63^, they appear to provide the region with the ability to rapidly conduct information to/from other sub-regions. While in a healthy state this evolutionary advantage serves a sophisticated basis for self-awareness and social cognition^17^, within a pathological state, this may manifest as a ‘disadvantage’ via atypical network engagement leading to an inability to sufficiently sustain engagement of the FPN nodes for hierarchical cognitive and executive processing. The SMG has also been termed a ‘hub region’ given its high connectedness within the neural networks attributed to the region’s larger layer III neurons^64^, which is considered as the primary layer for long-range cortico-cortical communication^65^. In our sample, concussed males had increased rs-FC between the temporal/opercular cortices and the SMG node of the SN. This is in sharp contrast to the reduced and localized FPN connectivity difference we observe in females across similar temporal regions. The SMG plays an important role in post sensory processing of salient stimuli for evaluating, categorizing, responding and decision making^66,67^ and is linked to verbal learning and memory. Our findings of increased rs-FC in the SMG appears to have a sex-based specificity towards males. In light of the varying cytoarchitecture of this relatively localized region and its functional importance with respect to attention processing, this dynamic will need to be explored further to better understand the functional impact of this shift in macroscale connectivity especially as it relates to sex-specific changes. Interestingly, reduced FC in the SMG has been reported in the behavioral variant of frontotemporal dementia (predominantly male sample) and is also a region that shows markers for neuroinflammation and reactive gliosis in former NFL players^68,69^. Previously, Popescu and colleagues^70^ have correlated cognitive performance with early (~ 200 ms) evoked cortical activity across a distributed network including the posterior STG and SMG; interestingly, also, in a sample of predominantly male participants with PCS.

While evidence examining sex-specific differences post-concussion is scarce, there appears to be some convergence across studies. In particular, Hsu and colleagues (2015) have reported similar interaction effects; mTBI females had persistent hypoactivation, across key frontal and parietal regions at a 6-week follow-up compared to males. Additionally, in 2018, there has been emerging support for a female “structural vulnerability”: (1) Dolle et al. have shown a sex-specific structural disadvantage in axonal microstructure that places female axons at greater risk of failure during similar trauma states, (2) Sollmann et al., have provided evidence of a susceptibility across global and large fiber tracts, and, more recently, (3) Rubin et al. have concluded that, despite similar exposures to heading in soccer, females exhibit more widespread evidence of white matter alteration when compared to men. These findings altogether support our hypothesis of a region-specific female “vulnerability” to injury in the FPN circuitry post concussion. Nevertheless, we also report a male-specific “vulnerability” to injury along similar cortical regions of interest albeit by way of a key node of the SN (SMG). We urge that the global generalization of concussion being a female or male vulnerability should be avoided. Our findings point to regional sex-specific vulnerabilities that are driven by different nodes of two large scale networks and future work should examine these vulnerabilities to better understand the organizational disruptions post-concussion as well as clinical relevance to rehabilitation.

### Strengths and limitations

The main strength of this study is the large sample size compared to any other study published to date exploring rs-FC in the PCS population. This advantage has enabled us to explore and interpret group-level results for both injury- and sex-based differences and we have utilized best practices^71–73^ to consider, collect characterize and communicate sex-based analyses in this study. Varied mechanisms of injury and time-lapsed since injury has allowed interpretation across a heterogeneous spectrum.

Contrary to previous studies^74–76^, we did not observe any group differences either within or between the DMN. We propose this may be due a number of factors; including temporal variability related to time-lapsed since injury, large age variances and/or the fact that 16% of our sample had experienced more than one concussion. Additionally, self-reported concussion and associated symptoms may be viewed as a subjective bias, although, the current diagnostic criterion for concussion is based on self-report.

## Conclusion

We explored underlying alterations in both intra- and inter-network connectivity while exploring sex-specific changes in rs-FC in long lasting PCS. We interpreted the results within the context of neural alterations in core networks that impair hierarchical processing in primary and association cortices thus potentially impairing behavior. We conclude that while males may be more prone to sustaining a brain injury, certain architectural vulnerabilities especially in the temporal cortices, may predispose females to injury more than males post concussion. Hierarchical processing pathways can be sex-specific largely due to underlying anatomical asymmetries between the sexes. The triple-network model provides a unique framework for characterizing neural alterations post-concussion.
